# Immune microenvironment of infantile hemangioma: cellular dynamics, molecular networks, and emerging immunomodulatory strategies

**DOI:** 10.3389/fimmu.2026.1908287

**Published:** 2026-08-27

**Authors:** Linyu Yang, Xue Gong, Yulang Xu, Kaizhi Zhang, Shanshan Xiang, Yunhan Zhang, Yi Ji

**Affiliations:** Division of Oncology, Department of Pediatric Surgery, West China Hospital of Sichuan University, Chengdu, China

**Keywords:** angiogenesis, immune microenvironment, immune–vascular crosstalk, immunomodulatory therapy, infantile hemangioma, macrophage polarization

## Abstract

Infantile hemangioma (IH), the most common vascular tumor of infancy, is characterized by a paradoxical natural history of rapid proliferation followed by spontaneous but often incomplete involution. Although dysregulated angiogenic signaling has long been considered central to IH pathobiology, an angiogenesis-only model is insufficient to explain its phase-dependent behavior, marked clinical heterogeneity, syndromic associations, or variable response to therapy. Emerging evidence instead supports the view of IH as a dynamically remodeled immune–vascular lesion driven by signaling crosstalk among endothelial cells, stem/progenitor cells, stromal elements, extracellular matrix, and immune populations. In this narrative review, we summarize the current knowledge of the immune microenvironment of IH across the proliferative and involution phases, focusing on macrophage polarization, mast cell maturation, dendritic and dendritic-like stromal populations, neutrophil-associated inflammatory signaling, adaptive immune constraints, lymphatic endothelial networks and extracellular matrix remodeling. We further discuss the molecular networks that shape this landscape, including VEGF- and HIF-1α-driven angiogenic programs, metabolic immunosuppression linked to glycolysis and lactate accumulation, and hormonal and immune checkpoint pathways that may contribute to immune tolerance and vascular persistence. Accordingly, we examine the immunological mechanisms of current therapies, particularly propranolol, corticosteroids, and mTOR inhibitiors, and highlight how refractory, segmental, syndromic, or ulcerated IH may reflect distinct immune states. Finally, we outline future directions for mechanism-based treatment, including immune microenvironment reprogramming, metabolic targeting, lesion-confined drug delivery, and biomarker-guided precision stratification. Together, these advances support a transition from empiric vascular suppression toward lesion-targeted immunomodulatory therapy in IH.

## 1 Introduction

Infantile hemangioma (IH) represents the most prevalent pediatric vascular tumor, affecting approximately 5% of newborns, with incidence rates ranging from 2% to 10% (1, 2). IH occurs more frequently in white, premature and low-birth-weight infants and has a striking female predominance (3, 4), with a female-to-male ratio of approximately 3:1 (5). Over the past four decades, the incidence of IH has increased steadily, possibly because of improved survival rates among preterm infants who carry a 40% elevated risk of developing IH (6, 7). Maternal factors associated with an increased incidence of IH include advanced age, multiple pregnancies, infertility treatments, *in vitro* fertilization, prenatal procedures (e.g., amniocentesis), complications (such as pre-eclampsia and placenta previa), first-trimester bleeding, and smoking (8–10).

**Figure 1:**
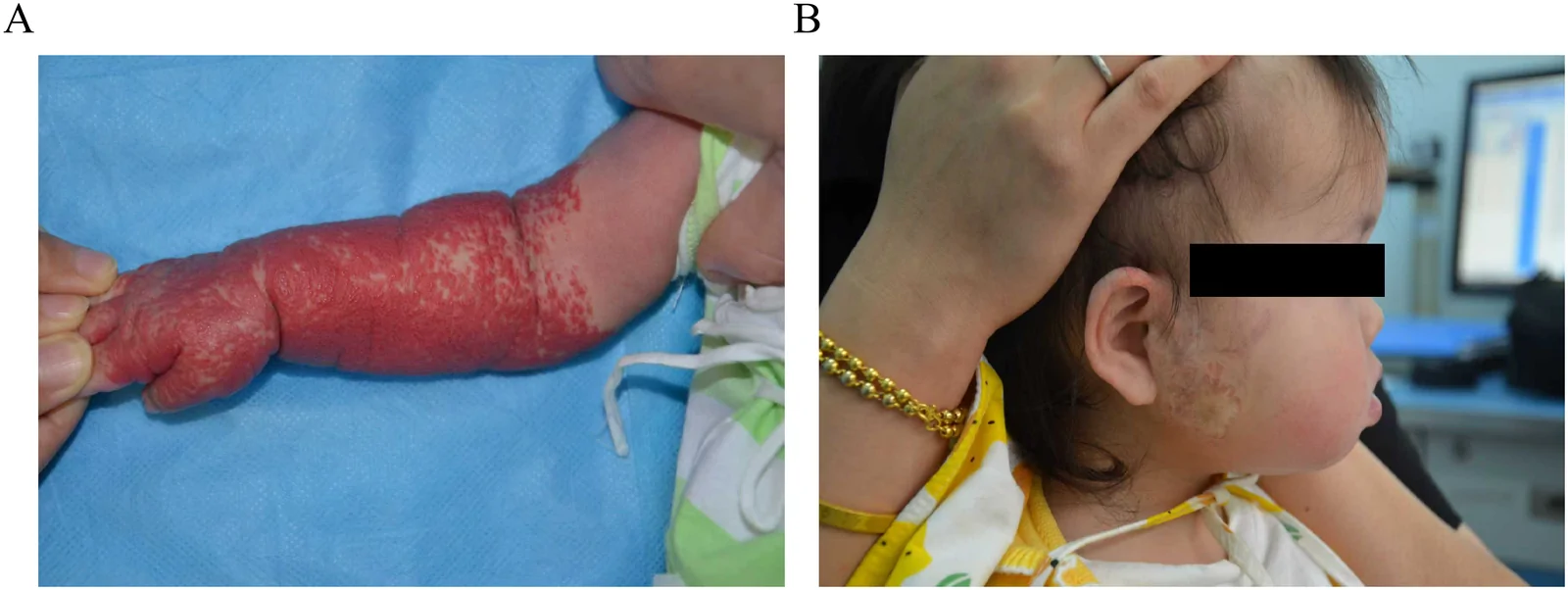
Representative clinical phenotypes across the heterogeneous course of IH. **(A)** Large segmental IH of the upper extremity, representing an extensive lesion phenotype that may show prolonged proliferation and require closer clinical monitoring. **(B)** Residual facial changes after IH involution, demonstrating that regression is often incomplete and may leave permanent fibrofatty and cutaneous sequelae.

Clinically, IH follows a unique and paradoxical life cycle characterized by rapid growth, usually completed by 9–12 months of age (7, 11), followed by slow and often incomplete involution over several years, with a fibrofatty residuum remaining in 50–70% of cases (12–14). Although the majority of IHs undergo spontaneous regression, a substantial subset exhibit aggressive growth, delayed involution, or clinically significant complications that necessitate early and active intervention (7, 15, 16). Prolonged proliferation is more common in large, segmental, and/or deep lesions, particularly those located in the head and neck region, and is associated with a greater risk of functional impairment and permanent disfigurement (**Figure 1**) (17).

**Figure 2:**
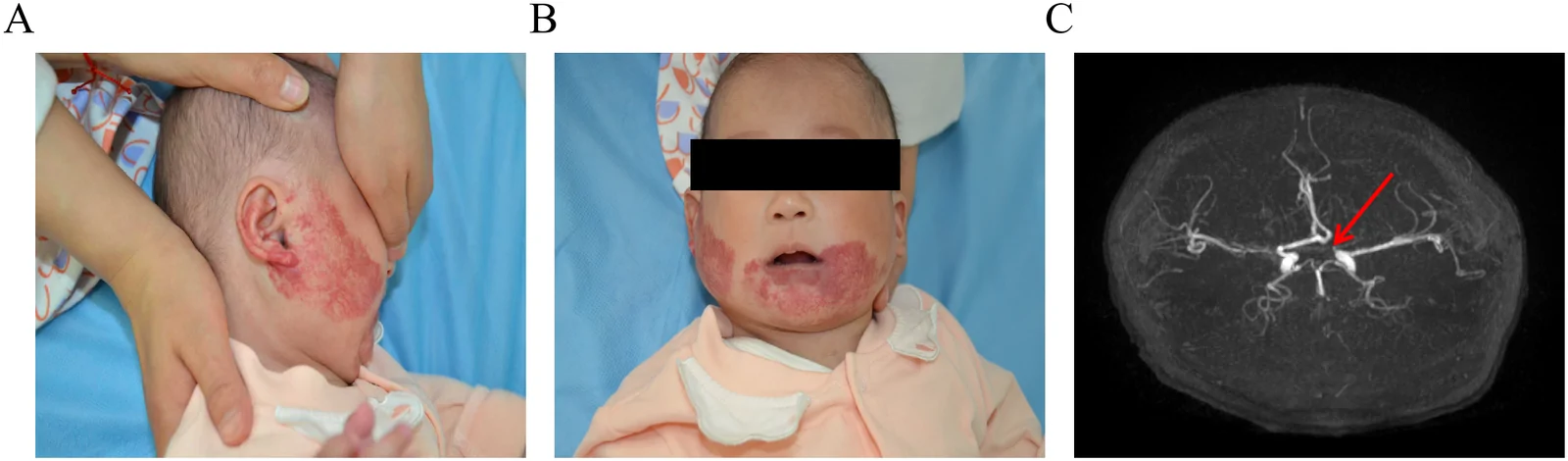
Segmental IH associated with PHACE syndrome. **(A, B)** Representative patient with a large segmental infantile hemangioma involving the head and neck region, a distribution pattern that is strongly associated with PHACE syndrome. **(C)** Brain magnetic resonance angiography demonstrating absence of the left middle cerebral artery, consistent with the cerebrovascular anomalies that may accompany PHACE syndrome.

**Figure 3:**
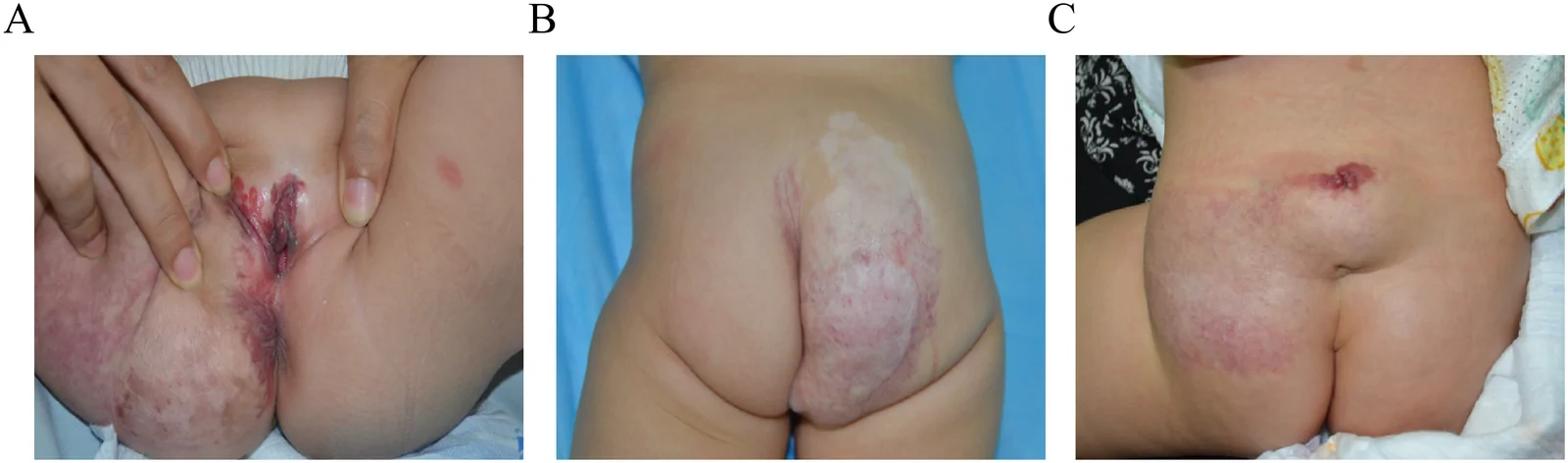
Clinical manifestations of LUMBAR syndrome. Representative clinical photographs of three different patients with LUMBAR syndrome, illustrating the heterogeneous distribution and morphology of segmental infantile hemangiomas involving the lumbosacral, gluteal, and perineal regions. **(A)** Extensive segmental infantile hemangioma involving the gluteal and perineal regions, accompanied by ulceration. **(B)** Broad segmental infantile hemangioma predominantly involving the unilateral gluteal and perineal region. **(C)** Segmental infantile hemangioma involving the lumbosacral and gluteal region with focal ulceration.

The anatomical distribution of IH is nonrandom and appears to correspond to developmental units or embryonic vascular territories rather than dermatomes or neural pathways (18–22). IH can be associated with serious complications depending on its type and location (23). Multifocal IHs, occurring in approximately 3.6% of cases, are associated with a significant risk of hepatic involvement when patients have more than five lesions (24, 25), which may be accompanied by potential hypothyroidism or rare intestinal issues (26). Segmental IHs, particularly large lesions in the head and neck or lumbosacral regions, are prone to ulceration and functional impairment, and are frequently linked to major structural anomalies through specific syndromes (9). Segmental IHs involving the head and neck region are strongly linked to PHACE syndrome (27, 28), a neurocutaneous disorder encompassing Posterior fossa malformations, Hemangioma, Arterial anomalies, Cardiac defects, Eye abnormalities, and ventral developmental defects (**Figure 2**) (28–31). Similarly, segmental IHs in the lumbosacral or perineal area in the lower body may indicate LUMBAR syndrome (32), characterized by Lower body IHs, Urogenital anomalies, Myelopathy, Bony deformities, Anorectal malformations, and Renal or arterial anomalies (**Figure 3**) (15, 33, 34). These syndromic associations highlight the intimate connections between IH, vascular patterning, and systemic developmental processes.

Current management strategies for IHs include pharmacotherapy, laser treatment, interventional procedures, and surgical excision. Propranolol, a non-selective β-adrenergic blocker, is the first-line and FDA-approved therapy and has dramatically improved clinical outcomes (35–38). Despite its efficacy, limitations persist, including potential adverse events, central nervous system penetration, and a rebound rate of up to 25% upon discontinuation (37–41). A long-term follow-up study revealed that 72.4% of patients with IHs who were treated with oral propranolol developed residual lesions, such as fibrofatty tissue and skin laxity, ultimately requiring surgical resection (17). These clinical challenges indicate that the mechanisms driving IH initiation, progression, and incomplete regression remain incompletely understood.

The pathogenesis of IH has been attributed to dysregulation of angiogenic signaling pathways, including those involving vascular endothelial growth factor (VEGF), hypoxia-inducible factor-1α (HIF-1α), angiopoietins, and estrogen-mediated signaling (42–44). While these canonical mechanisms account for endothelial proliferation and vascular expansion, they fail to fully explain several defining features of IH, including its tightly programmed transition from rapid proliferation to spontaneous involution, its marked interlesional heterogeneity, and its strong association with developmental syndromes and perinatal risk factors.

Studies have shown that immune and immune-mediated inflammatory events may contribute to the pathogenesis of IH (45). Immune cells dynamically interact with hemangioma-derived endothelial cells (HemECs) and stem cells (HemSCs), pericytes, fibroblasts, and extracellular matrix components (ECM), collectively shaping a distinct immune microenvironment (46–48). Notably, new classes of therapeutics, such as immune checkpoint blockade (ICB), explicitly target the tumor microenvironment (TME) rather than the tumor cells themselves, offering a novel approach to targeted therapy.

This narrative review synthesizes the current knowledge of the immune microenvironment in IH, delineates its dynamic remodeling during disease progression and involution, examines the immunological basis of existing therapies, and discusses emerging immunomodulatory strategies with potential clinical relevance.

## 2 Components of the IH immune TME

**Figure 4:**
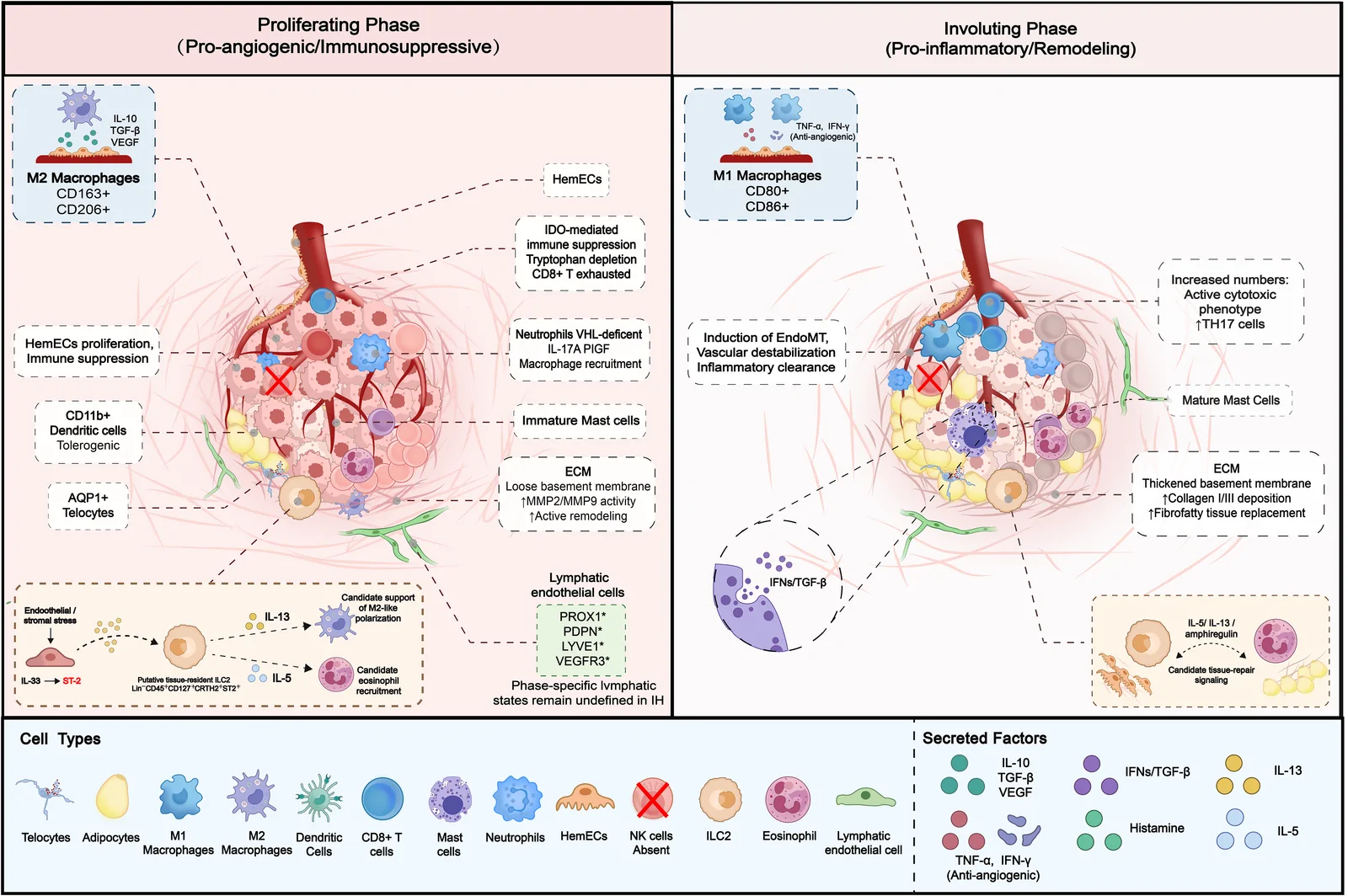
Dynamic immune–vascular remodeling of infantile hemangioma across proliferative and involuting phases. During the proliferative phase, the IH microenvironment is characterized by a predominantly pro-angiogenic and immunosuppressive state. M2-like macrophages (CD163^+^CD206^+^) produce immunoregulatory and angiogenic mediators, including IL-10, TGF-β, and VEGF, and support HemEC proliferation and vascular persistence. IDO-mediated tryptophan depletion may constrain CD8^+^ T-cell activity, whereas neutrophil-associated inflammatory signaling, including the VHL–HIF/PlGF and IL-17A pathways, may further promote macrophage recruitment and pathological vascular growth. Immature mast cells, tolerogenic CD11b^+^ dendritic cells, AQP1^+^ telocytes, and an actively remodeled extracellular matrix characterized by a relatively loose basement membrane and increased MMP2/MMP9 activity further contribute to the proliferative niche. Putative tissue-resident group 2 innate lymphoid cells (ILC2s) are included as a candidate component of type 2 immunity: endothelial or stromal stress may induce IL-33/ST2 signaling, leading to IL-5- and IL-13-associated eosinophil recruitment and M2-like macrophage polarization. These ILC2-related pathways remain hypothetical in IH. Lymphatic endothelial cells, characterized by markers such as PROX1, PDPN, LYVE1, and VEGFR3, are also incorporated as a potential immune-regulatory compartment, although their phase-specific states in IH remain undefined. During involution, the microenvironment shifts toward a pro-inflammatory and remodeling-associated state, with increased M1-like macrophage activity (CD80^+^CD86^+^), enhanced cytotoxic and Th17-associated immune responses, vascular destabilization, EndoMT, and inflammatory clearance. Mature mast cells may contribute IFNs and TGF-β, whereas extracellular matrix remodeling is characterized by basement-membrane thickening, increased collagen I/III deposition, and progressive fibrofatty replacement. ILC2–eosinophil-associated IL-5, IL-13, and amphiregulin signaling is shown as a candidate tissue-repair pathway during involution. Collectively, the figure illustrates the transition of IH from a proliferative, angiogenic, and immune-tolerant microenvironment toward an inflammatory, remodeling, and regression-associated state, while highlighting ILC2 and lymphatic pathways as emerging hypotheses requiring direct validation in IH.

The TME of IH represents a highly dynamic ecosystem in which immune cells, vascular components, and stromal elements collectively regulate lesion growth and regression. Rather than exerting static pro- or antiangiogenic effects, immune cell populations within IH undergo profound quantitative and functional remodeling across the proliferative and involution phases (**Table 1**) (49, 50). This temporal plasticity enables distinct immune subsets to play dual and sometimes opposing roles, alternately promoting angiogenesis, immune tolerance, inflammatory clearance, and tissue reconstruction (**Figure 4**) (50, 51).

**Table 1:** Immune and stromal components in the infantile hemangioma microenvironment.

Cell type	Key markers	Prevalent in	Major functions	Impact on
Macrophages (TAMs)	CD68, CD163, CD206	M2: proliferative phase; M1:involuting phase	M2 promotes angiogenesis and HemSC endothelial differentiation; M1 induces EndoMT and inflammation	Regulate angiogenesis and drive phase transition
Mast cells	Tryptase, chymase, CD117	Increase in early involution	Release VEGF, FGF-2, histamine (proliferation); release IFNs and TGF-β (involution)	Dual role in angiogenesis and regression
Dendritic cells	HLA-DR, CD68, DC-SIGN	Both phases	Antigen presentation, immune surveillance, vascular niche interaction	May regulate immune recognition and vascular morphogenesis
Neutrophils	CD66b, MPO	Early proliferative phase	Produce VEGF, PlGF, IL-17A; activate inflammatory signaling	Amplify angiogenic and inflammatory microenvironment
Group 2 innate lymphoid cells	Lin−CD45+CD127+CRTH2+, GATA3, ST2	Not directly characterized	Produce IL-5 and IL-13; recruit eosinophils; potentially regulate M2 macrophages and tissue repair	Hypothesized phase-dependent immune–vascular remodeling
CD8^+^ T cells	CD3, CD8	Increase in involution	Cytotoxic immune response	Promote lesion regression
Th17 cells	CD4, IL-17	Involution	Promote inflammatory remodeling	May contribute to regression process
B cells	CD20, CD79a	Both phases	Humoral immune response	Possible role in immune modulation
NK cells	CD56	Rare/absent	Cytotoxic immunity	Their absence may contribute to impaired cytotoxic immune surveillance and vascular persistence.
Extracellular matrix	Collagen I/III/IV, laminin, MMP2/9	Dynamic	Structural support and signaling	Regulates angiogenesis and fibrofatty replacement

### 2.1 Macrophages

Macrophages are among the most abundant immune cells within the IH microenvironment and serve as key regulators of angiogenesis, immune tolerance, and tissue remodeling (52). Within IH lesions, HemECs and HemSCs secrete chemoattractants such as M-CSF, CCL2, and CCL5, driving robust macrophage recruitment and differentiation into tumor-associated macrophages (TAMs) (53). In most cases, elevated numbers of TAMs in the TME are associated with tumor progression, metastasis, poor prognosis, and shorter survival in patients with various cancers, such as bladder cancer (54), breast cancer (55), and pancreatic cancer (56). TAMs are important cellular components involved in the pathogenesis of IH (45, 49). In general, TAMs are divided into two subtypes: M1 (classically activated macrophages) and M2 (alternatively activated macrophages) (57).

M1 macrophages are characterized by proinflammatory and immunostimulatory properties, including the production of TNF-α and IFN-γ, and have been implicated in endothelial injury, immune activation, and lesion regression in IH. These cytokines can directly kill cancer cells and activate the adaptive immune system, rendering M1 macrophages crucial components in cancer therapy (58). M1, but not M2, macrophages drive endothelial-to-mesenchymal transition (EndoMT) in IH, thereby facilitating vascular destabilization and lesion involution (59). In contrast, M2 macrophages predominantly exert proangiogenic and immunosuppressive effects, supporting hemangioma growth, immune evasion, and vascular stabilization during the proliferative phase. Consequently, suppressing M2 macrophages or reprogramming them into the M1 phenotype represents a common therapeutic strategy for solid tumors (60). M2 macrophages are increased in proliferating versus involuting IH (49). Although IL-4 and IL-13 are frequently invoked as M2-polarizing signals, their cellular sources in IH remain undefined. In addition to conventional T helper 2 (Th2) cells, tissue-resident group 2 innate lymphoid cells (ILC2s) should be considered potential contributors to IL-13 production, whereas their contribution to IL-4 expression appears to be more context dependent (61). Xia et al. demonstrated that hypoxia-induced ATF4 expression promotes proliferative IH progression by enhancing M-CSF-mediated M2 macrophage recruitment and polarization (62). Upon implantation with HemSCs in mice, the endothelial differentiation of HemSCs *in vitro*, was found to be promoted, and M2 macrophages translated this effect into a proangiogenic phenotype marked by elevated microvessel density and delayed adipocyte formation (50). In a reciprocal feedback loop, HemSCs confer ferroptotic resistance to macrophages through the upregulation of GPX4 expression, a process that seems essential for sustained IH progression (63). MIR4435-2HG in M2 macrophage-derived exosomes promotes the progression of IHs and enhances the proliferation, migration, invasion, and angiogenesis of HemECs through direct binding to HNRNPA1 (64). In pancreatic ductal adenocarcinoma, the presence of M2 macrophages and TIE2-expressing monocytes is correlated with reduced overall and recurrence-free survival (65). Furthermore, in triple-negative breast cancer, M2 macrophages promote angiogenesis by upregulating PCAT6 expression, which in turn modulates VEGFR2 expression (66). Additionally, cutaneous squamous cell carcinoma-related research has indicated that M2 macrophages facilitate angiogenesis via the circ_TNFRSF21/miR-3619-5p/ROCK2 regulatory axis (67).

### 2.2 Mast cells

Mast cells (MCs), as tissue-resident sentinels of the innate immune system (68, 69), are pivotal regulators within the TME of IH. Their abundance, maturation status, and functional output are highly dynamic, enabling them to play a complex dual role in both the proliferation and involution of IH through the release of a wide array of mediators (70). MCs originate from bone marrow–derived progenitors that circulate in the peripheral blood and subsequently migrate into peripheral tissues, where they undergo terminal differentiation (71–74). The number and activity of MCs fluctuate significantly across the different pathological stages of IH (75): MCs are less abundant and predominantly immature during the proliferative phase, whereas their numbers peak, and they appear highly active and mature during the early involution phase (76, 77). This temporal shift suggests that MCs contribute differently to IH pathobiology depending on the microenvironmental context.

Estradiol (E2) has been identified as a key chemoattractant that facilitates the migration of MC precursors into IH tissue (73, 77). Upon localization, MCs can be activated through several distinct pathways (78): 1) ligand binding to the Mas-related G-protein-coupled receptor X2 (MRGPRX2); 2) activation of ion channels by benzoyl ATP; and 3) physical contact with activated T-cell membranes and released microvesicles (70). These activation signals converge on intracellular calcium influx and downstream ERK1/2 signaling, culminating in MC degranulation and mediator release (79). Activated MCs exert bidirectional control over IH progression through the secretion of various modulators (51). During the proliferative phase, MC-derived factors—including tryptase, chymase, histamine, type VIII collagen, VEGF, and FGF-2—promote HemEC proliferation and neovascularization and contribute to ECM remodeling (80, 81). These proangiogenic activities position MCs as important amplifiers of vascular expansion within the early IH microenvironment. As IH transitions into the involution phase, the functional profile of MCs shifts toward an antiangiogenic and proregressive phenotype. They secrete anti-angiogenic factors including interferon (IFN-α, IFN-β, and IFN-γ) and transforming growth factor-beta (TGF-β), which downregulate pro-angiogenic factors and inhibit endothelial cell mitosis (82, 83). In parallel, the expression of apoptogenic proteins such as clusterin/apolipoprotein J by MCs during involution may contribute to the regression process (76).

### 2.3 Dendritic cells

Dendritic cells (DCs) represent a specialized antigen-presenting cell population within the immune microenvironment of IH and exhibit distinctive phenotypic and spatial features that distinguish IH from both malignant tumors and inflammatory vascular disorders (84). Early histopathological studies identified a unique perivascular cell population in IH lesions that displayed a dendritic morphology and high expression of HLA-DR and CD68, with partial expression of DC-SIGN, but lacking classical Langerhans cell markers such as CD1a, Langerin, and DC-LAMP (84). This unique phenotype (HLA-DR+ CD68+ DC-SIGN±) suggests that these cells belong to a specialized monocyte–macrophage–related antigen-presenting lineage rather than conventional DC subsets (85). Notably, these DC-like cells are intimately associated with the vascular wall of IH lesions, forming a structural and functional interface between immune surveillance and vascular morphogenesis. Similar perivascular antigen-presenting cells are present in the dermal microvascular unit of normal skin but are conspicuously absent in the perivascular niches of cutaneous melanoma metastases (86, 87). Their distinctive distribution pattern even supports the hypothesis that IH may originate from “vasculogenesis” rather than classical “angiogenesis” (88). Recent research provides a new perspective on the role of DCs in IH. Integrated bioinformatics analysis and experimental validation revealed significant enrichment of activated DCs in the IH microenvironment. More importantly, the cell surface marker CD11b has been identified and validated as a potential biomarker for these activated DCs in IH, with immunofluorescence confirming its significant upregulation in IH tissues (89). Telocytes (TCs) are DCs that form a distinctive peripheral layer in IH, with highly specific expression of AQP1. AQP1-positive TCs are critical for the responsiveness of IH to propranolol (90). These findings provide novel insights into DC-related mechanisms in IH, identifying activated DCs as a compelling therapeutic targets for further development.

### 2.4 Neutrophils

Neutrophils are being increasingly recognized as active contributors to the immune TME of IH rather than transient bystanders of inflammation. During the early proliferative phase of IH, neutrophil infiltration is elevated, reflecting the activation of innate immune pathways while simultaneously suggesting the presence of immune escape mechanisms that permit unchecked vascular expansion (91). Experimental evidence from murine models has established a direct pathogenic role for neutrophils in hemangioma formation. Genetic inactivation of the von Hippel–Lindau (VHL) tumor suppressor in neutrophils promotes pathological vascular proliferation through activation of the HIF-2α–placental growth factor (PlGF) axis. Bone marrow transplantation experiments further demonstrated that VHL-deficient neutrophils are sufficient to initiate hemangioma-like vascular lesions, highlighting the role of neutrophils as active drivers rather than secondary responders in IH pathogenesis (92). In a liver fibrosis model, Tan et al. reported that neutrophils are the predominant IL-17A–producing cells in the liver and that their expression is regulated by the transcription factor RORγt. IL-17A not only directly activates hepatic stellate cells to promote fibrosis but also enhances ECM production through ERK/p38 MAPK signaling—a mechanism likely relevant to stromal remodeling in IH (93). In addition to producing proangiogenic factors such as VEGF and PlGF, neutrophils amplify the inflammatory and angiogenic microenvironment via the IL-17A/IL-17RA axis, recruiting additional immune cells such as macrophages to sustain vascular abnormalities and tumor growth (94). Clinical observations further underscore the relevance of neutrophil-driven inflammation in hemangioma-associated pathology. Elevated neutrophil-to-lymphocyte ratios have been correlated with inflammatory complications and postoperative pain following hemangioma interventions, suggesting that systemic neutrophil activation mirrors local immune dysregulation within IH lesions (95). Together, these findings position neutrophils as innate immune amplifiers that are coupled with hypoxia sensing, cytokine production, and immune cell recruitment to sustain pathological vascular remodeling in IH.

### 2.5 ILC2s: a candidate regulator of type 2 immunity

ILC2s are lineage-negative, tissue-resident lymphocytes enriched in barrier organs, including the skin. They respond rapidly to epithelial or endothelial alarmins, particularly IL-33, IL-25, and thymic stromal lymphopoietin, and generate type 2 mediators (96). ILC2s are major innate sources of IL-5 and IL-13 across several tissues. IL-5 supports eosinophil accumulation, whereas IL-13 directly or indirectly promotes alternative macrophage activation and tissue repair (97). In visceral adipose tissue, genetic depletion of ILC2s reduces eosinophils and alternatively activated macrophages, whereas IL-33 expands this cellular axis.

Direct evidence for ILC2s in IH is currently lacking (98). Nonetheless, an ILC2–macrophage interaction is biologically plausible because IH arises in the skin, undergoes phase-dependent tissue remodeling, and contains prominent macrophage and mast-cell populations. During proliferation, ILC2-derived IL-13 can reinforce M2-like macrophage programs and vascular stabilization; during involution or ulceration, ILC2-derived amphiregulin and interactions with eosinophils may influence tissue repair (97). Conversely, pathological metabolites may limit ILC2 abundance. In melanoma, tumor-derived lactate suppresses ILC2 proliferation, cytokine production, and survival, indicating that scarcity within a lesion does not necessarily imply biological irrelevance (99).

### 2.6 Adaptive immune cells: T lymphocytes, B lymphocytes, and the absence of natural killer cells

Adaptive immune cells in IH appear to be present but are functionally constrained, suggesting that inefficient immune surveillance may contribute to lesion persistence. Lesion-infiltrating T lymphocytes are detected in most IH lesions and are predominantly composed of CD3^+^CD8^+^ cytotoxic T cells, whereas CD4^+^ helper T cells are relatively rare. These CD8^+^ T cells infiltrate the lesion stroma, indicating preserved migratory capacity but limited effector function. During the proliferative phase, local immunosuppressive mechanisms restrict effective T-cell-mediated clearance. Elevated indoleamine 2, 3-dioxygenase (IDO) expression depletes tryptophan, suppresses T-cell proliferation, and delays immune-mediated regression (100). As IH progresses toward involution, CD8^+^ T cells and Th17 cells become enriched, whereas γδ T cells decline; moreover, IL-6, IRF1 and IL32 may participate in shaping this transition (101).

B cells have been detected in both proliferating and involuting IH lesions as interstitial CD20^+^ and CD79a^+^ cells, but their abundance appears to decrease during involution and their functional role remains largely undefined (102). Subsets of B and T lymphocytes have also been reported to express NPYR1, suggesting a possible neuro–immune interface in IH, although the biological relevance of this observation requires further validation (46).

In contrast to B and T cells, natural killer (NK) cells appear to be notably absent from the immune infiltrate of IH. Despite the presence of multiple immune cell populations, CD56^+^ NK cells are undetectable across the proliferative and involution stages (100). This finding indicates that mature, cytotoxic NK cells are likely not recruited into the IH lesion, or are present in numbers that are too low to be detected by immunohistochemistry.

Collectively, adaptive immune cells in IH exhibit preserved infiltration but constrained cytotoxic function, while the absence of NK cells highlights a permissive immune environment that favors vascular persistence. These features further support the concept of IH as an immune-modulated vascular lesion rather than a purely proliferative endothelial disorder.

### 2.7 Lymphatic vasculature as an immuno-regulatory compartment

The lymphatic vasculature is an active immuno-regulatory compartment rather than merely a drainage system. Lymphatic endothelial cells maintain tissue-fluid balance, establish chemokine gradients, transport antigens, guide CCR7+ dendritic-cell migration to draining lymph nodes, and regulate lymphocyte activation and tolerance (103). In melanoma, the expression of lymphatic markers is correlated with immune infiltration, whereas the loss of dermal lymphatic vessels reduces local cytokine expression and leukocyte accumulation while altering the efficacy of transferred cytotoxic T cells (103). Blockade of lymphangiogenesis in peritumoral adipose tissue also promotes IL-6 accumulation and enrichment of alternatively activated macrophages, linking lymphatic dysfunction to macrophage remodeling and angiogenesis (104).

In IH, the lymphatic-vessel density, drainage function, and lymphatic endothelial-cell state across proliferative and involution phases remain largely undefined. This gap is relevant because IH combines vascular hyperpermeability, inflammatory remodeling, ulceration in a subset of lesions, and eventual fibrofatty replacement. Altered lymphatic drainage can influence interstitial pressure, antigen and cytokine transport, immune-cell entry or egress, and the spatial organization of macrophages and DCs (98).

### 2.8 Stromal and ECM regulation of immune–vascular remodeling

The ECM serves as a structural scaffold for blood vessels and actively participates in the initiation, proliferation, and involution of IH. Multiple studies have highlighted the dynamic changes in the ECM composition across different phases of IH development. During the proliferative phase, IHs exhibit active angiogenesis and endothelial cell proliferation. Tan et al. reported the consistent presence of collagen type IV, the β_2_ chain of laminin, and perlecan in the vascular basement membrane (105). Notably, short-chain collagen type VIII was detected extracellularly and intracellularly within MCs, suggesting a role in early angiogenesis. Concurrently, increased expression of matrix metalloproteinase 2 (MMP2) and MMP9 facilitates ECM remodeling and endothelial migration. In the involution phase, significant alterations in ECM composition occur. Compared with proliferating lesions, involuted IHs have significantly higher levels of collagen type III and laminin, particularly in the perivascular basement membrane (106). These changes coincide with fibrofatty tissue deposition and vascular maturation. Furthermore, studies have shown that miR-206 promotes ECM accumulation by targeting DNMT3A, leading to the upregulation of COL2A1 and aggrecan expression and the suppression of MMP2/MMP9, thereby inhibiting the pathological behavior of HemECs (107). In the involution phase, further ECM remodeling is observed, characterized by thickened basement membranes, increased deposition of interstitial collagens (types I, III, and V), and replacement of vascular lumina with fibrofatty tissue (105). These structural changes are closely associated with clinical regression.

## 3 Molecular networks shaping the IH immune microenvironment

**Figure 5:**
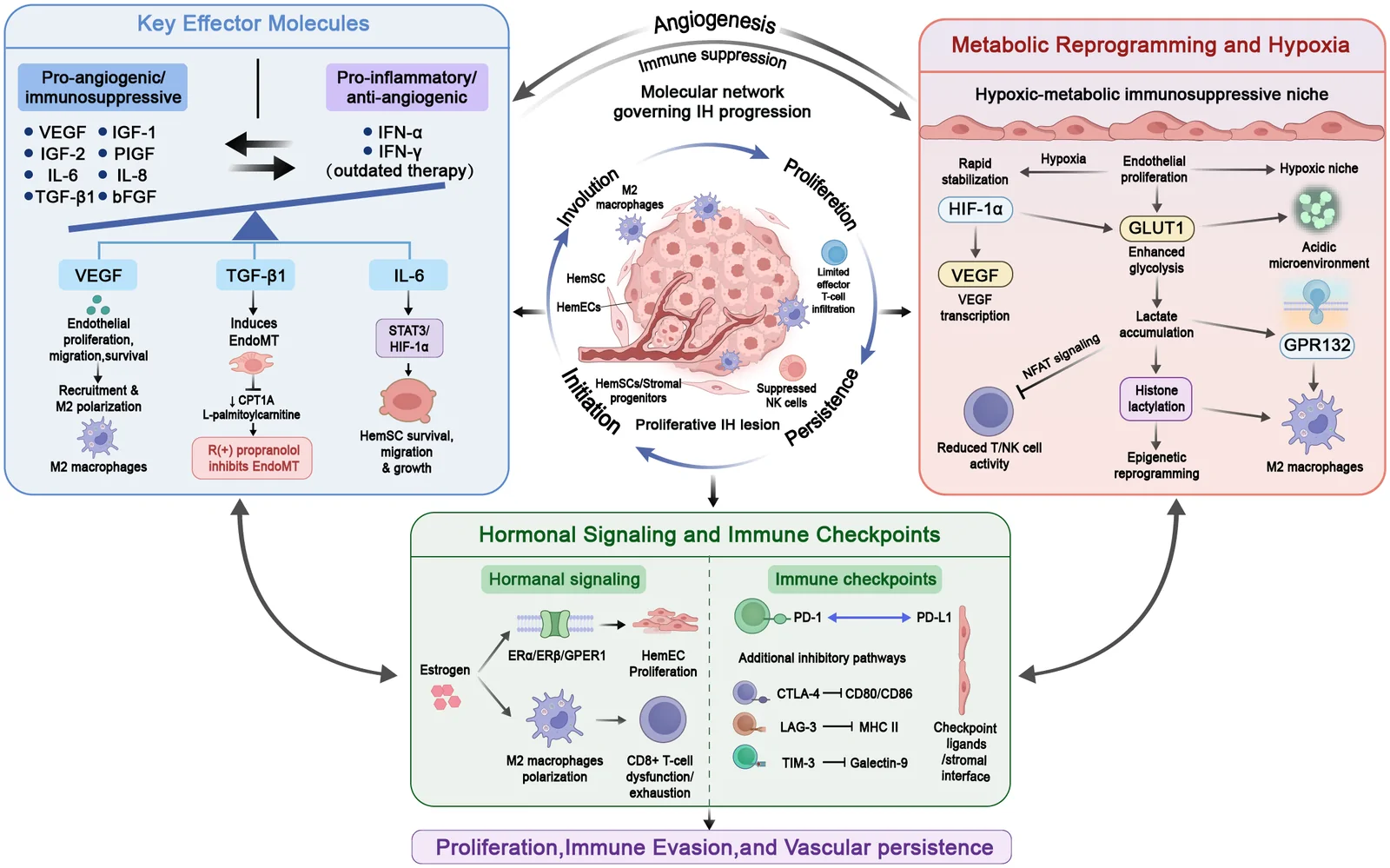
Molecular networks shaping immune–vascular states in IH. VEGF, IGF-1 and TGF-β1 promote endothelial proliferation, macrophage recruitment and M2-like polarization, thereby supporting angiogenesis and immune tolerance. During involution, inflammatory mediators, including interferon-associated signals, may promote immune activation, endothelial destabilization and vascular regression. Hypoxia, enhanced glycolysis and lactate accumulation may reinforce immune suppression and M2-like macrophage programs. Candidate metabolic–immune mediators, including NFAT- and GPR132-related pathways, may link lactate signaling to immune-cell reprogramming. Hormonal signaling and checkpoint-associated pathways, including PD-1-related, CTLA-4-related and TIM-3-related signals, may contribute to immune restraint and vascular persistence.

The immune TME of IH is not static but is a dynamic “ecosystem” controlled by the balance of diverse molecular mediators. These mediators form a complex communication network, encompassing not only functional cytokines with direct cellular actions but also microenvironmental features shaped by metabolic reprogramming, hormonal signals, and critical immune checkpoint molecules. Together, they form a sophisticated regulatory network that governs the entire life cycle of IH, from initiation and proliferation to involution (**Figure 5**).

### 3.1 Cytokine and angiogenic networks

Clinical and translational studies have demonstrated that circulating and tissue-level cytokine profiles vary significantly with IH activity and burden. Proangiogenic and immunomodulatory factors—including insulin-like growth factor-1 (IGF-1), IL-6, IL-8, PIGF, CCL5, and TGF-β1—are dynamically regulated during disease progression (108). After 5 years, another clinical study revealed that the level of 4 of the 20 cytokines (bFGF, IFN-γ, IGF-I, and TGF-β1) were greater in IH patients with multiple lesions than in those with a single lesion (109). Serum GLUT1, IGF-2, and VEGF-A levels were positively correlated with disease severity in patients with IH (110). Among these mediators, VEGF is central to the proliferative IH microenvironment. VEGF is abundantly secreted by hemangioma endothelial cells, where it acts in autocrine and paracrine manners to potently promote endothelial cell proliferation, migration, and survival (111), and it also actively recruits macrophages and polarizes them toward an immunosuppressive M2 phenotype (50). TGF-β1 promotes the progression of IH by inducing EndoMT through the downregulation of CPT1A and its metabolite L-palmitoylcarnitine, whereas R(+) propranolol can inhibit this process (112). TGF-β also effectively suppresses the activity of T cells and NK cells, and M2 macrophages can secrete; however, these potential mechanisms have not yet been definitively validated in IH, suggesting an important direction for future research. IL-6 promotes the survival and growth of IH by increasing the viability and migration of HemSCs while inhibiting apoptosis, primarily through the STAT3/HIF-1α signaling pathway (113). IFN-α is used to treat severe IH. It can induce significant shrinkage of the hemangioma mass within a relatively short period and alleviate complications caused by rapid tumor growth. However, owing to its potential risk of spastic paralysis, it is now rarely used in clinical practice (109).

### 3.2 Hypoxia and immunometabolic remodeling

Metabolic cues constitute a fundamental and underappreciated layer of immune regulation within the IH microenvironment (114). Rapid endothelial proliferation during the proliferative phase generates localized hypoxia, stabilizing HIF-1α and driving the transcription of VEGF and glucose transporters such as GLUT1 (115, 116). This hypoxia-driven metabolic reprogramming tightly couples angiogenesis with altered immune function. Enhanced glycolytic flux within IH lesions leads to the accumulation of lactate and acidification of the local microenvironment. Lactate is not merely a metabolic byproduct but also a signaling molecule: it impairs T/NK cell function by inhibiting NFAT signaling (117) and promoting M2 polarization via G-protein coupled receptors (e.g. GPR132) (118). Metabolic suppression may also affect ILC2s. In melanoma, tumor-derived lactate reduces activated ILC2 proliferation, cytokine production, and survival, whereas a reduction in LDHA increases the intratumoral ILC2 abundance. Thus, a low ILC2 frequency in pathological tissue may reflect metabolic exclusion rather than lack of functional relevance (99). More recently, the discovery of histone lactylation has provided a mechanistic link between metabolism and epigenetic immune reprogramming. Lactate-derived histone modifications reshape macrophage transcriptional programs, consolidating an immunosuppressive phenotype and stabilizing the proangiogenic microenvironment (119).

### 3.3 Hormonal and immune checkpoint signaling: hijacking immune restraint

The marked female predominance of IH underscores the central role of estrogen. Estrogen not only directly promotes the proliferation of hemangioma cells via its receptor (GPER1) (120) but also acts as an immune “editor” (121, 122). FSH signaling may also contribute to IH progression, as FSHR is enriched in proliferating IH endothelium and tracks with the early growth phase of the lesion (123). In melanoma, males receive a greater benefit from ICB than females do (124–126). This variation is noted because ERα activity in the stromal cells of the microenvironment can promote the accumulation of M2-type macrophages or induce macrophages polarization toward the M2 type, which leads to dysfunction and exhaustion of CD8+ T cells, resulting in tumor progression (124). This immune skewing may be a key mechanism underlying female predisposition and immune evasion in IH.

Immune checkpoints act as intrinsic brakes on immune activation to ensure self-tolerance. During the proliferative phase, these checkpoints are upregulated, enabling immune escape. The PD-1/PD-L1 axis is the most classic immune checkpoint pathway. Anti-PD-1 therapy in metastatic malignant melanoma has resulted in significant response. HemECs highly expressed of PD-1 but were negative for PD-L1. Moreover, the infiltrated lymphocytes within the IH were positive for PD-1 (127). In addition to PD-1/PD-L1, a complex synergistic inhibitory network exists. CTLA-4 inhibits T-cell activation by binding to CD80/CD86; LAG-3 negatively regulates T cells by binding to MHC class II molecules (128); and TIM-3 induces apoptosis in Th1 cells upon binding to its ligand galectin-9 (129).

Together, these findings suggest that hormonal signaling and immune checkpoint pathways co-opt the physiological mechanisms of immune tolerance to establish a permissive microenvironment that favors vascular persistence. These hijacked “brake” signals represent attractive targets for therapeutic modulation, particularly in refractory or poorly involuting IH.

## 4 Immunological mechanisms of existing therapies

The natural history of IH is a direct manifestation of the active transition of its immune microenvironment from a “proangiogenic/immunosuppressive” state to a “proinflammatory/vaso-involutive” state (59, 100). During the proliferation phase, a microenvironment dominated by molecules such as VEGF, bFGF, TGF-β, and IL-10 directly stimulates the massive proliferation of vascular endothelial cells and actively constructs an immunosuppressive/tolerant niche by recruiting and “educating” macrophages into the M2 phenotype and expanding Tregs. These microenviroment-induced changes allow the nascent vascular mass to evade immune surveillance and achieve rapid growth. Upon entering the involution phase, this balance is disrupted. A proinflammatory network centered on IFN-γ, TNF-α, and IL-1β, among others, begins to dominate. The infiltration of cytotoxic T cells and NK cells increases, and macrophages polarize toward the proinflammatory M1 phenotype. Simultaneously, MMPs are activated, initiating the degradation of the vascular basement membrane and ECM remodeling. Therefore, involution is not merely a simple “cessation of growth” but an immune system-mediated, highly programmed process of “inflammatory clearance and tissue reconstruction.”

### 4.1 The potential immunological mechanisms of existing therapies

**Figure 6:**
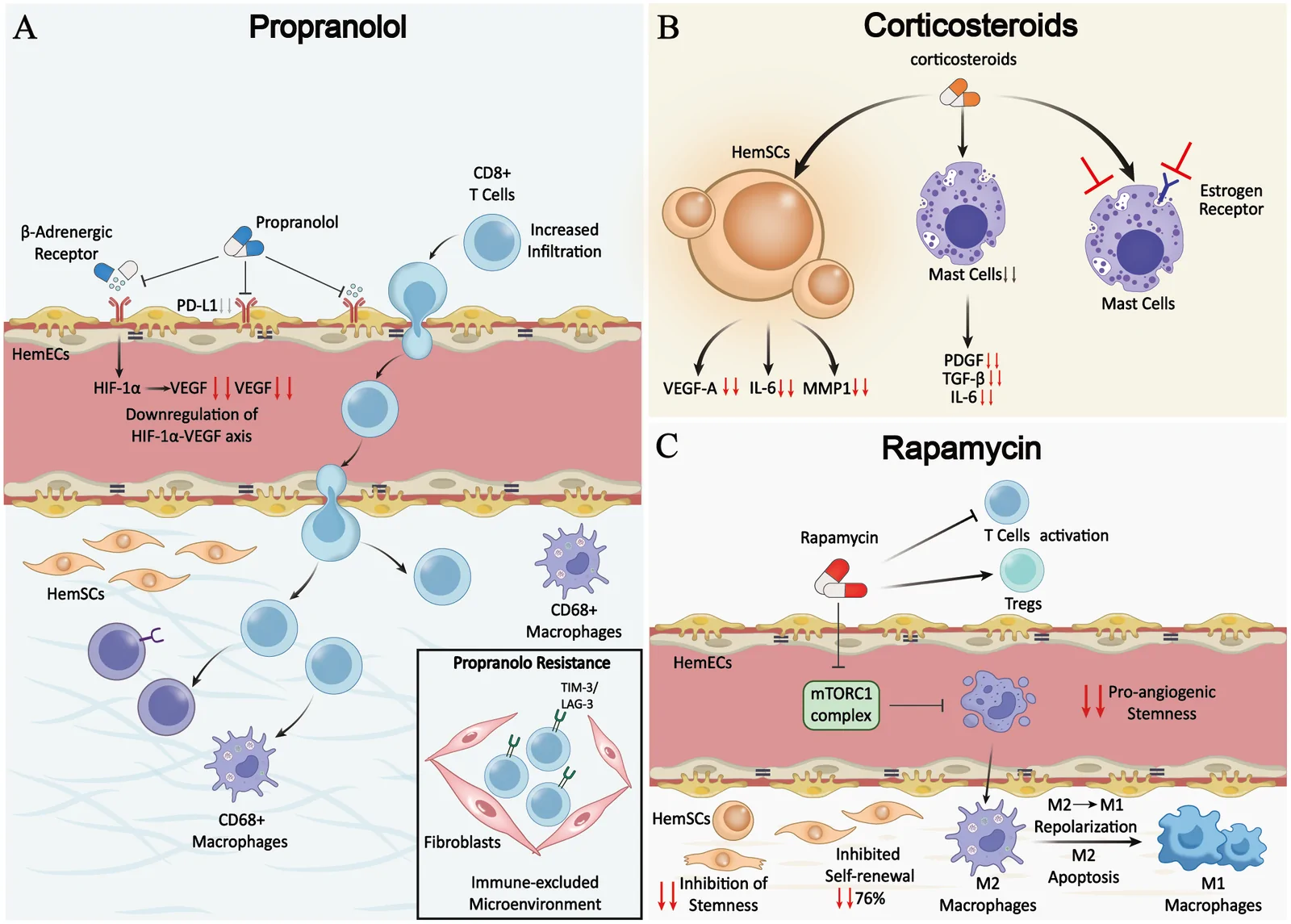
Therapeutic modulation of immune–vascular signaling in IH. **(A)** Propranolol attenuates β-adrenergic signaling and downregulates the HIF-1α–VEGF axis in IH-associated vascular cells. This reduces endothelial proliferation and may indirectly reshape immune–vascular communication. Its broader effects may include partial attenuation of macrophage-supported angiogenic and immunosuppressive circuits. **(B)** Corticosteroids suppress VEGF-A, IL-6 and MMP1 production and may influence mast-cell-associated inflammatory and angiogenic responses. They may also interfere with hormone-associated signaling, although the relevance of this effect in IH requires further validation. **(C)** mTOR inhibition suppresses HemSC self-renewal, endothelial proliferation and metabolic signaling. It may also modulate macrophage polarization and immune-cell activation, linking therapeutic response to both vascular and immunometabolic remodeling.

Drugs traditionally thought to function primarily through direct vasoconstriction or apoptosis induction likely depend profoundly on “reprogramming” the immune microenvironment (**Figure 6**).

#### 4.1.1 Propranolol: a “Resetter” of the immune microenvironment?

Propranolol, the first-line therapy for IH, was initially believed to act primarily through vasoconstriction and direct inhibition of endothelial proliferation (130–132). Recent studies suggest that the efficacy of propranolol extends far beyond simple beta-blockade (133). It may indirectly affect VEGF-driven immunosuppression by downregulating the HIF-1α–VEGF axis. In ovarian and breast cancers, propranolol can reduce immunosuppression caused by stress (especially when PD-L1 levels are elevated), and when it is used in combination with PD-L1 inhibitors, it can better activate the immune system to attack tumor cells and reduce metastasis (134). A phase II randomized controlled trial in breast cancer demonstrated that short-term preoperative administration of propranolol downregulated intratumoral mesenchymal gene expression and the activity of prometastatic transcription factors (such as NF-κB and AP-1), but increased the tumor infiltration of CD68+ macrophages and CD8+ T cells (135).

Rebound growth after propranolol discontinuation is noted in 19-25% of patients (136). Our prospective study reported that the initiation of propranolol treatment after 3 months of age and an IH location on the nasal tip, breast area, or diaper area were independent risk factors for a poor response at 6 months (137). The mechanisms underlying propranolol resistance (nonresponse or rebound) remain unclear, but an immunological perspective offers new insights. Resistant lesions may possess a more robust immunosuppressive barrier, such as: (1) compensatory upregulation of alternative immune checkpoint molecules (e.g., TIM-3 and LAG-3); (2) the presence of a unique, drug-irreversible T-cell exhaustion profile; and (3) the formation of an “immune-excluded” microenvironment maintained by specific fibroblast or macrophage subsets, preventing T-cell infiltration. These speculations provide a theoretical basis for exploring combination immunotherapies (e.g. propranolol combined with topical PD-1 inhibitors).

#### 4.1.2 Corticosteroids: the classic broad-spectrum immunosuppressants

Before the advent of propranolol, systemic corticosteroids were the mainstay of IH treatment (138–141). Their therapeutic effects arise from a combination of direct antiangiogenic activity and broad immunosuppressive actions. The mechanism underlying the treatment of in treating IH is complex, and includes direct antiangiogenic effects (inhibiting VEGF) and broad immunosuppression. Dexamethasone suppressed VEGF-A production by HemSCs *in vitro*, reduced vasculogenesis *in vivo* and simultaneously decreased the generation of IL-6 and MMP1 (142). Hansan el al reported that steroid therapy increased the number of MCs in IH, and inhibited the expression of platelet-derived growth factors, such as IL-6, TGF-β, cytochrome b, and other angiogenesis-related factors (143). Additionally, corticosteroids antagonize estrogen signaling, which may contribute to their efficacy given the hormone-dependent immune skewing observed in IH (144). However, their significant side effects with systemic use limit their long-term application, highlighting the need to develop more targeted immunomodulators.

#### 4.1.3 Rapamycin (and its analogs): precision modulation targeting the mTOR pathway

Sirolimus has demonstrated effectiveness in treating IH, particularly in patients who are resistant to conventional treatments such as corticosteroids and propranolol (145, 146). As an mTOR inhibitor, it disrupts the mTOR signaling pathway, which regulates cell growth, proliferation, and vasculogenesis (147). In IH, sirolimus inhibits the self-renewal and differentiation of HemSCs, leading to vascular regression (148). The mTOR pathway is a central hub that integrates growth factor, nutrient, and energy signals, and is crucial for the activation of both immune cells and vascular endothelial cells (149). Rapamycin, by inhibiting mTORC1_activity, directly inhibits hemangioma endothelial cell proliferation (150). Immunologically, it suppresses T cell activation and proliferation, while potentially affecting macrophage differentiation and function, and has some ability to modulate Tregs (151–154). Experiments have shown that rapamycin can weaken the renewal ability of HemSCs by 76%, reduce their differentiation potential (148), and induce M2 apoptosis but not M1 apoptosis. Moreover, it can induce macrophages to transform into M1 macrophages (155). These combined vascular and immune effects position mTOR inhibition as a precision-modulating strategy capable of reshaping the IH microenvironment. For complex, refractory IHs, the topical or systemic use of rapamycin to target this key pathway shows considerable promise.

### 4.2 Refractory/complex IH: does a unique immune escape architecture exist?

Not all IHs follow the typical natural history. Some segmental, large-area, or poorly involuting IHs are classified as “refractory/complex” (15, 156, 157). Predictive factors for IH regrowth include discontinuation of propranolol before 9 months of age, a deep IH component, and female sex (41). However, the underlying mechanisms driving regrowth in this patient subgroup are not yet fully understood and the mechanism of action of propranolol has yet to be elucidated (1, 158). From an immunological standpoint, these cases may represent a distinct subtype of IH with fundamental differences in their immune microenvironments (49). For instance, they might: (1) exhibit an immune-cold-like state with relatively limited T-cell infiltration; (2) show persistent local immunosuppressive signaling potentially related to intralesional cellular heterogeneity; and (3) be associated with innate immune dysregulation, potentially including complement-related pathways. Utilizing technologies such as single-cell sequencing and spatial transcriptomics to compare the immune landscape of typical IH versus that of refractory IH is a the future direction for identifying key differences and driving mechanisms (159, 160).

### 4.3 Ulcerated IH: The “wound healing” mode of immune imbalance

Ulceration is the most common complication of IH and is generally thought to result, at least in part, from tissue ischemia and surface necrosis during rapid proliferation (161, 162). From an immunological perspective, the ulcerated area is a battlefield where acute inflammation and abnormal repair coexist (163). Ulceration disrupts the original microenvironmental balance, exposing subcutaneous tissue and triggering a strong acute inflammatory response dominated by neutrophil and M1 macrophage infiltration, which is aimed at clearing necrotic tissue and fighting infection (164–168). However, under the influence of persistent proangiogenic signals (e.g. VEGF from the surrounding hemangioma) and potential bacterial colonization, the wound healing process is prone to dysregulation, shifting toward pathological repair characterized by fibrosis and chronic inflammation, leading to pain, slow healing, and scarring (169–172). Therefore, managing ulcerated IH requires not only controlling proliferation and infection but also changing the local immune response from “destructive inflammation” to “constructive repair.”

## 5 potential therapeutic strategies and future directions

The paradigm shift from conceptualizing IH as a purely vascular anomaly toward recognizing it as an immune-mediated pathology has opened new avenues for therapeutic intervention. Drawing on recent advances in the treatment of vascular malformations, future strategies should prioritize the repolarization of the TME and precision drug delivery (173).

### 5.1 Reengineering the immune microenvironment

Given the critical role of macrophage plasticity in IH, strategies that shift macrophages away from a pro-angiogenic M2-like state toward a regression-associated M1-like program may hold therapeutic promise (49, 50, 59). Small molecules or antibodies targeting the CSF-1/CSF-1R axis can deplete proangiogenic TAMs or reprogram them toward an antitumor phenotype (174, 175). Type 2 immunity and IL-33–ST2–ILC2 signaling warrant cautious exploration as future immunomodulatory axes. In an ILC2/eosinophil-rich environment, IL-33 may support organized immune activation and tissue repair; in a Treg- or suppressive myeloid-cell-rich environment, the same signal may reinforce immunosuppression and remodeling (96). Likewise, VEGF-C/VEGFR3-directed manipulation is premature until lymphatic density and drainage are defined in IH, because lymphatic vessels may support both immune priming and immune resolution (98). Furthermore, considering PD-1 expression on infiltrating lymphocytes, topical immune checkpoint inhibitors (ICIs) could be explored (127). Unlike systemic ICIs used in oncology, topical administration of ICIs (e.g. imiquimod or novel PD-1 antibodies) might induce local regression in refractory superficial IHs without systemic toxicity, effectively “releasing the brakes” on the host’s natural involution mechanisms (176–179).

### 5.2 Metabolic targeting

Hypoxia-driven glycolysis and lactate accumulation are likely to contribute to the immunosuppressive microenvironment of proliferative IH (180, 181). In this context, targeting metabolic checkpoints, including glycolytic enzymes or lactate transporters such as MCT1/4, may help disrupt the acidic niche that impairs cytotoxic lymphocyte function and supports M2-like macrophage polarization (117, 118, 182–184). This “metabolic immunotherapy” approach reframes metabolic inhibition not merely as antiproliferative (185), but also as a lever to restore immune competence within the lesion.

### 5.3 Advanced drug delivery systems

A major translational bottleneck in IH immunotherapy is not merely target discovery, but also the ability to deliver bioactive agents safely, efficiently, and selectively to the lesion. Poor transdermal penetration limits conventional topical therapies, whereas systemic administration raises safety concerns in infants (186, 187). Advanced delivery systems offer a solution by confining immunomodulation to the lesion (**Table 2**) (188).

**Table 2:** Potential therapeutic strategies and emerging approaches targeting the immune microenvironment of IH.

Platform	Most relevant clinical scenario	How it would be used (route/frequency)	Clinical benefit hypothesis (vs current care)	Immune microenvironment angle	Key safety/monitoring points	Superficial	Deep	Ulcerated	Refractory	Translational barrier/next step
Microneedle patch	Small-to-moderate **superficial IH** needing faster response or improved adherence	Office-or caregiver-applied patch; **intermittent dosing** (e.g., every few days rather than daily topical)	Higher local exposure; less caregiver burden, reduce need for systemic β-blocker in borderline cases	Lesion-confined microdosing may bias macrophages toward **pro-involution** state and dampen pro-angiogenic inflammation	Local irritation, pain, secondary infection risk; confirm minimal systemic exposure (vitals if β-blocker delivered)	✓	△	△	✓	Demonstrate real-world feasibility; standardized patch time, depth consistency, and pediatric tolerability
Microneedle + long-acting depot	**Deep/mixed IH** where sustained local effect indeed patients who cannot tolerate systemic therapy	Short application → **weeks-long local effect**; likely clinic-supervised early	“Induction + maintenance” without daily dosing; potential reduction in relapse after stopping therapy	Sustained exposure supports durable macrophage-state shift and longer immune remodeling window	Depot persistence; delayed skin reaction; systemic spillover (blood levels)	△	✓	△	✓	Build dose-interval guidelines; prove predictable clearance and repeat dose safety in juvenile models
Microneedle + trigger (e.g., light/heat)	**Refractory focal lesions** needing escalation; highly selected cases	MN delivery then brief in-clinic trigger, episodic sessions	Rapid debulking and perfusion shut down; may res cue resistant lesions without systemic immunotherapy	Controlled “lesion editing” may generate danger signals that reset local immune tone and break tolerance	Burn/scar risk; pain; pigment change; strict trigger calibration	△	△	—	✓	Define pediatric-safe trigger window and standardized protocols; show benefit outweighs scarring risk
Targeted nanoparticles(local/lesion-directed)	**Deep IH** or complex lesions where cell-selective delivery may matter	Local or semi-local delivery (ideally via MN/hydrogel embedding); schedule depends on formulation	Improve efficacy at lower dose; reduce systemic exposure: enable RNA/precision drugs not feasible as free drug	Cell-selective delivery (HemEC vs TAM) enables **immune-vascular co-remodeling)**	Biodistribution/persistence; liver/kidney labs; long-term follow-up	△	✓	△	✓	Establish pediatric Biodistribution/clearance; scalable manufacturing; lesion-specific targeting evidence
Hydrogel dressing (immunomodulatory wound dressing)	**Ulcerated IH** (pain, exudate; infection risk)	Dressing applied to ulcer surface; daily/alternate-day depending on wound	Faster pain relief and healing reduce infection; adjunct to β-blocker to prevent recurrence	Creates a local immune-conditioning niche; suppresses excessive inflammation and supports repair-associated macrophage programs	Contact dermatitis; infection surveillance; avoid over-granulation; monitor ulcer healing trajectory	△	—	✓	△	Need IH-ulcer-specific clinical endpoints and protocols; avoid inadvertently promoting angiogenesis in proliferative lesions
Bioactive matrices (vesicle-based/local biologic)	Ulcerated or post-treatment remodeling; selected refractory cases in trials	Local dressing or injection-like application depending on format	Accelerate repair and microenvironment; Potentially reduce scarring	Paracrine-style immune-education to promote pro-resolving macrophage states	Standardization/consistency; immunogenicity; regulatory complexity	△	△	✓	△	Define potency/identity assays and batch QC; early-phase safety in pediatrics
Hybrid systems (MNs + nanoparticles/MNs + hydrogel)	Patients needing both depth access and prolonged control (e.g., **deep + ulcerated**, refractory)	MN for placement + matrix for retention; individualized schedule	Combine rapid local control with long-term remodeling; reduce relapse	Enables spatiotemporal immune programming (prime → maintain)	Complexity; skin burden; adherence	✓	✓	✓	✓	Keep design minimal; p redefined Go/No-Go criteria (PK, clinical response, safety, usability)

Bold values indicate the most clinically relevant or distinctive features of each therapeutic platform. “✓” indicates high or primary applicability to the corresponding clinical scenario, “△” indicates potential or conditional applicability requiring careful patient selection and/or further validation, and “—” indicates that the approach is generally not applicable to that clinical scenario.

Microneedle-based systems can bypass the stratum corneum and deposit drugs directly into the epidermal–dermal compartment, thereby markedly increasing intralesional exposure and reducing systemic absorption (186). In IH, dissolvable microneedles have already been used to enhance the local delivery of timolol and bleomycin (186, 189), and can be further adapted for the sustained release of immunoregulatory agents or combined with functions such as vascular embolization (190–192).

Targeted nanoparticles provide a complementary strategy for deeper, more complex, or treatment-refractory lesions (187). Ligand-modified carriers targeting lesion-associated vascular markers such as VEGFR2 may improve the delivery of small molecules (193), nucleic acid therapeutics, or exosome-mimetic cargos to HemECs, HemSCs, or potentially lesion-associated macrophages (194). In preclinical IH studies, nanocarriers have improved the pharmacokinetics and therapeutic efficacy of propranolol and have also shown promise for nucleic acid or exosome-based cargo delivery (187, 195–197), supporting their potential as platforms for immune-directed or immune-vascular combination therapy.

Future efforts should focus on multifunctional local platforms that integrate targeting, sustained release, and safety control, particularly for deep, segmental, or propranolol-refractory IH.

### 5.4 Personalized medicine and biomarkers

Refractory, segmental, and syndromic IHs (including PHACE-associated lesions) may represent distinct immunogenomic subsets rather than simply severe variants of the same disease (198). Integrating of single-cell RNA sequencing and spatial transcriptomics into clinical studies may allow the stratification of patients based on immune architecture, potentially revealing phenotypes analogous to immune-hot, immune-cold, or immune-excluded states (159, 160, 199).

Such stratification aligns with broader advances in vascular anomaly research and supports genotype- and immune-informed therapeutic selection (200, 201). Ultimately, precision IH therapy will likely require matching immunomodulatory strategies to the specific immune and metabolic landscape of each lesion.

## 6 Conclusion

IH is increasingly understood not as a purely endothelial overgrowth disorder but as a dynamically regulated immune–vascular ecosystem (49, 50, 59, 127). Across its natural history, the lesion-level microenvironment transitions from a proangiogenic, immune-tolerant state—characterized by hypoxia-driven signaling, immunosuppressive macrophage programs, and checkpoint-like restraints—to a proinflammatory, remodeling-permissive state that supports vascular regression and fibrofatty replacement. This immune-centered framework helps reconcile the hallmark paradox of explosive early growth followed by spontaneous involution in IH (202), and it provides a coherent explanation for clinical heterogeneity, syndromic associations, and the variable depth-dependent behavior of lesions.

While propranolol remains the cornerstone therapy, non-response, rebound growth, and complicated phenotypes (136, 203) (e.g., deep/segmental or ulcerated IH) suggest that durable clinical control often requires more than vascular suppression—namely, effective reprogramming of local immune architecture. Moving forward, the most actionable opportunities lie in linking mechanistic immune insights (macrophage plasticity, metabolic immunosuppression, and checkpoint restraint) with pediatric-compatible (49, 59, 127, 185), and lesion-confined delivery platforms and biomarker-guided stratification. By integrating spatial/single-cell immune profiling with precision delivery (microneedles, nanoparticles, and bioactive matrices) (1, 160), future IH therapeutics may shift from empirical management toward mechanism-based, lesion-targeted immunomodulation that accelerates regulated involution while maintaining systemic safety.
